# Depth Discrimination in Diffuse Optical Transmission Imaging by Planar Scanning Off-Axis Fibers: INITIAL Applications to Optical Mammography

**DOI:** 10.1371/journal.pone.0058510

**Published:** 2013-03-14

**Authors:** Jana M. Kainerstorfer, Yang Yu, Geethika Weliwitigoda, Pamela G. Anderson, Angelo Sassaroli, Sergio Fantini

**Affiliations:** Department of Biomedical Engineering, Tufts University, Medford, Massachusetts, United States of America; University of California, Irvine, United States of America

## Abstract

We present a method for depth discrimination in parallel-plate, transmission mode, diffuse optical imaging. The method is based on scanning a set of detector pairs, where the two detectors in each pair are separated by a distance δ*D_i_* along direction **δ** ***D*** *_i_* within the *x*-*y* scanning plane. A given optical inhomogeneity appears shifted by α_i_ **δ** ***D*** *_i_* (with 0≤ α_i_ ≤1) in the images collected with the two detection fibers of the *i*-th pair. Such a spatial shift can be translated into a measurement of the depth *z* of the inhomogeneity, and the depth measurements based on each detector pair are combined into a specially designed weighted average. This depth assessment is demonstrated on tissue-like phantoms for simple inhomogeneities such as straight rods in single-rod or multiple-rod configurations, and for more complex curved structures which mimic blood vessels in the female breast. In these phantom tests, the method has recovered the depth of single inhomogeneities in the central position of the phantom to within 4 mm of their actual value, and within 7 mm for more superficial inhomogeneities, where the thickness of the phantom was 65 mm. The application of this method to more complex images, such as optical mammograms, requires a robust approach to identify corresponding structures in the images collected with the two detectors of a given pair. To this aim, we propose an approach based on the inner product of the skeleton images collected with the two detectors of each pair, and we present an application of this approach to optical *in vivo* images of the female breast. This depth discrimination method can enhance the spatial information content of 2D projection images of the breast by assessing the depth of detected structures, and by allowing for 3D localization of breast tumors.

## Introduction

Near-infrared spectroscopy (NIRS) and diffuse optical imaging (DOI) employ visible and near-infrared light (typically over the wavelength range 650–1,000 nm) that achieves a penetration depth of several centimeters into biological tissue. As a result, NIRS and DOI can be used to investigate and image macroscopic tissue volumes, with applications such as functional brain imaging [1], muscle oximetry [2], and optical mammography [3], [4]. The latter application benefits from the sensitivity of NIRS and DOI to important breast tissue chromophores, namely oxy-hemoglobin, deoxy-hemoglobin, water, and lipids, and leads to the discrimination of benign and malignant breast tumors [5], [6], and monitoring response to neoadjuvant chemotherapy [7]–[10]. More specifically, optical mammography has shown its potential in detecting angiogenesis [11], hypoxia [12], [13], and collagen content as a measure of increased breast tissue density [14], which are all relevant physiological parameters for the diagnosis and characterization of breast cancer [15]–[17].

To enhance the spatial information content of DOI, it is desirable to achieve spatial resolution in three dimensions. This affords (1) the capability of assigning the spatial location of detected tissue lesions, and (2) the discrimination of tissue structures that may be overlapping in 2D projection images. In optical mammography, 3D spatial reconstructions have been obtained using circular arrangements of source and collection optical fibers around the breast by applying image reconstruction methods for Diffuse Optical Tomography (DOT) [18]–[20], and with parallel scanning approaches [5]. A full 3D spatial reconstruction is a complex task that suffers from the fact that the inverse diffuse imaging problem is ill-posed, from the non-linearity of the optical sensitivity function, and from calculations that are typically time consuming and computationally intensive. Two-dimensional planar projection imaging uses tandem scanning of a set of source and collection optical fibers on opposite sides of the slightly compressed breast (i.e. in a transmission geometry). This approach allows for a high spatial sampling rate (in the order of 1–2 mm) of optical data as well as high spectral resolution (in the order of 1 nm over a wavelength range of several hundred nm) when a spectrometer is used. The 2D planar projection imaging approach has been shown to extract fine spatial details in the optical images. For example, blood vessels can be found by taking full advantage of their large intrinsic optical contrast in tissue by using a second-derivative algorithm [21]. Going a step further, depth discrimination may be achieved in 2D planar projection imaging by combining data collected with two detector optical fibers that are offset with respect to each other over the scanning plane [22], [23]. We indicate the offset with the vector **δ** ***D***, whose magnitude (δ*D*) is the distance between the two offset detector fibers, and whose direction is defined by the line joining the two detector fibers. The challenging task of depth discrimination in this approach is to pair a given inhomogeneity in the image collected with the first detector with the corresponding inhomogeneity in the image collected with the second detector (the positions of the inhomogeneity in the two images are shifted by αδ*D*, with 0≤ α ≤1). If the optical images are dominated by one inhomogeneity or by a few inhomogeneities separated by more than δ*D* from each other, such task of pairing corresponding structures in the on-axis and off-axis images is straightforward and one can translate the offset parameter α into the depth of the corresponding inhomogeneity [22]. If, instead, the optical images contain several structures, one possible approach is to superimpose the two detector images [23], [24], in a fashion that is conceptually similar to geometric tomography and digital tomosynthesis. However, the diffusive nature of light propagation in tissues, as opposed to the mostly directional propagation of x-rays in tissues, accounts for a much stronger degrading effect from scattering on the combined two detector optical images versus x-ray digital tomosynthesis.

In this work, we propose a novel approach to the problem of pairing detected structures so that they can be assigned an offset parameter α and a corresponding depth *z*. The idea is to find a measure of correlation between structures that appear in the images collected with the two detectors, and are separated by no more than the optical fibers offset δ*D*. Such measure of correlation should not be based solely on the spatial dependence of the intensity perturbation associated with a given inhomogeneity along one specific direction. In fact, in diffuse optics such spatial dependence is dominated by the background optical properties rather than by the size and optical properties of the inhomogeneity. A more robust approach makes full use of the 2D spatial dependence of the transmitted intensity perturbation over the *x-y* scanning plane, which also reflects the shape of the inhomogeneity, especially for extended tubular structures such as blood vessels. Consequently, we base our pairing of structures in the two images on their 2D shape in the *x-y* scanning plane, which reflects their particular shape as projected onto the *x-y* plane. This approach takes advantage of the high spatial sampling rate (0.5 mm^−1^) of our 2D projection imaging collection [25], and of the enhanced identification and display of spatially distributed optical inhomogeneities with a second-derivative algorithm [21]. The offset parameter α assigned to a detected inhomogeneity is the one that maximizes the inner product between an intensity data matrix centered around the inhomogeneity in the first image, and a set of corresponding intensity matrices that are shifted along **δ** ***D*** by increments equal to the pixel size, in the second image. We have demonstrated the effectiveness of this depth assessment approach over a total sample or tissue thickness of 4.0 cm (Monte Carlo simulations), 6.5 cm (phantoms tests), and 5.5 cm (*in vivo* breast imaging). In the following sections, we describe the theoretical basis and the inner product algorithm for the proposed depth assessment approach, Monte Carlo simulation results, and the depth-assessment results obtained in tissue-like phantoms and in a human subject.

## Methods

### 2.1 Ethics Statement

The human subject examined was recruited from a large imaging study on optical mammography, which includes healthy volunteers as well as patients with breast cancer. Optical mammography is a non-invasive imaging method, which uses near-infrared light for imaging the breast. The study was approved by the Institutional Review Board at Tufts University. Written informed consent was obtained from the subject before performing the imaging session.

### 2.2 Basic Principles

**Figure 1:**
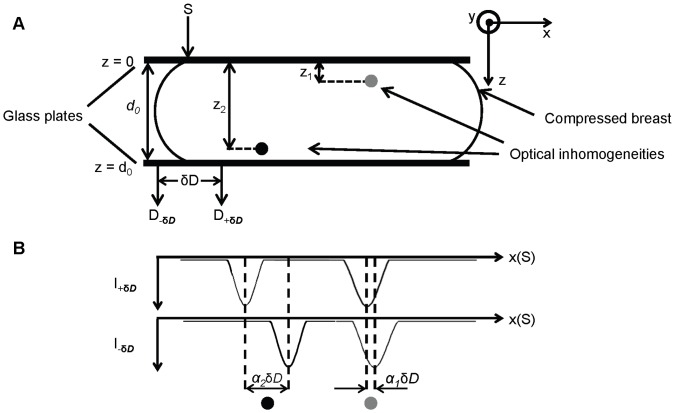
Basic principles of the approach to depth discrimination for parallel-plate, transmission imaging of the breast. In panel (A), D_-**δD**_ is the detection fiber offset along –**δD**, and D_+**δD**_ is the detection fiber offset along +**δD**. The distance between the two fibers is δD. Panel (B) shows the transmitted intensity profiles (I_+*δD*_ and I_-*δD*_) for both detectors as they are scanned along the **δ** ***D*** direction. The two optical inhomogeneities located at different depths *z*_1_ and *z*_2_, respectively, appear shifted along **δ** ***D*** by different amounts α_1_δ*D* and α_2_δ*D.*

The basic principle of depth discrimination is illustrated in Figure 1 for the case of a slightly compressed breast placed in between two parallel glass plates. The glass plates define the *x-y* plane over which the source fiber (S) and two detector fibers are scanned, while *z* is the depth coordinate of the optical inclusions, which may assume values between 0 and the plate separation (d_0_). Figure 1A shows two detection fibers that are separated by δ*D* and are both off-axis with respect to the source fiber, one shifted along -**δ** ***D*** (*D_-_*_**δ*D***_) and the other shifted along +**δ** ***D*** (*D_+_*_**δ*D***_). In the case of Figure 1, the two detection fibers are offset along *x* so that **δ** ***D*** = δ*D
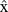
*. In this article, we use subscripts to indicate the positive (*D_+_*_***δD***_) or negative (*D_-_*_**δ*D***_) offset of each detector along a given direction **δ** ***D***, and a zero subscript (*D_0_*) indicates a detector optical fiber that is on-axis with respect to the source fiber. For all experiments, two off-axis detectors (*D_+_*_**δ*D***_ and *D_-_*_**δ*D***_) have been used. The only exception is the *in vivo* study, where we used one on-axis and one off axis detector.

The way in which the depth of a detected object affects the transmitted intensity perturbations measured with the two detectors is illustrated in Figure 1B. Two optical inhomogeneities located at different depths *z*_1_ and *z*_2_, respectively, appear shifted along **δ** ***D*** by different amounts α_1_ **δ** ***D*** and α_2_ **δ** ***D*** (where the shift is defined as the vector given by the position of the inhomogeneity in the *D_-_*_δ*D*_ image minus the position in the *D_+_*_δ*D*_ image). Figure 1B shows the transmitted intensity profiles for both detectors as they are scanned along the **δ** ***D*** direction (which coincides with the direction of 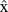 in Fig. 1). The fact that objects located at different depths result in different values (α_1_ and α_2_) of the offset parameter α, is the basis for the method proposed here.

**Figure 2:**
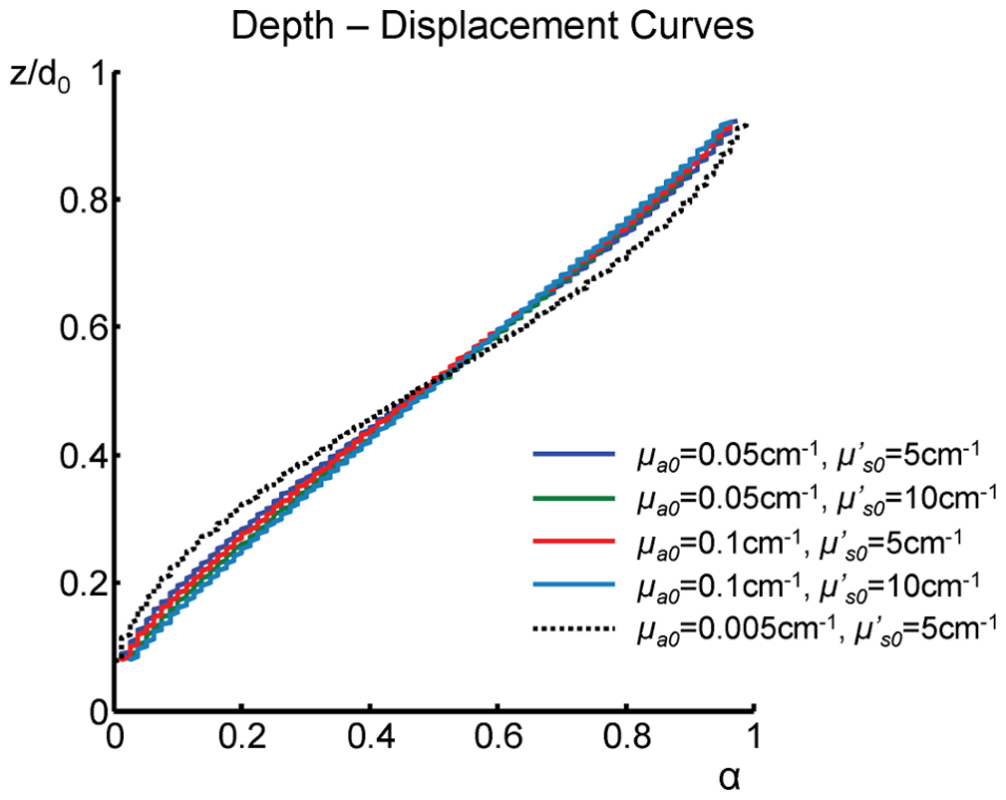
Depth curve. The measured shift α is translated into the depth z/d_0_ by using the relationship between α and z/d_0_ obtained using diffusion theory. The depth curves shown here are based on different background optical properties, as indicated in the figure.

The relationship between the depth *z* of an optical inhomogeneity and the offset parameter α is determined by the features of light propagation in turbid media and it is shown in Figure 2 for various optical properties of the medium. From a measurement of α and knowledge of *d_0_*, the curves of Figure 2 can be used to find the depth *z*. The curves of Figure 2 were obtained by using a first-order perturbation approach within diffusion theory in the infinite medium geometry. Note that, in the case of a single defect, the relationship derived within first order perturbation is also confirmed by higher order perturbation theory, which describes more realistic perturbations present in the human breast. Also, the same relationship between *z*/*d_0_* and α is found regardless of the specific position of the two detection fibers as long as the projection of the source fiber falls between them. In our diffusion-based computations, a single point-like absorption perturbation is embedded in a turbid medium of thickness *d*_0_. The calculations were carried out for a “unitary” perturbation, for which the product of the volume *(*V) and absorption contrast (Δµ_a_) is *V*Δµ_a_ = 1 mm^2^. Perturbation theory is used to generate 2D projection images on the *x-y* plane with two detection optical fibers for a depth *z* of the inhomogeneity varying over the full range of the slab thickness. By plotting the depth divided by the slab thickness (*z*/*d*_0_) versus the computed values of α, one finds the depth curve (shown in Fig. 2) that allows for the translation of a measured offset parameter α into the corresponding depth *z*. The various curves in Figure 2 differ in the background optical properties. Figure 2 illustrates that the shape of the depth curve is only weakly sensitive on the background optical properties over the absorption coefficient range of 0.05 to 0.1 cm^−1^ and reduced scattering coefficient range of 5 to 10 cm^−1^, which mimics a broad range of soft tissues. In that range, the maximum discrepancy between the curves was found to be <4%. For a much lower absorption coefficient of 0.005 cm^−1^, which corresponds to water absorption at 690 nm, a larger discrepancy up to ∼7% was found (Fig. 2 black dotted line).

We observe that this method of depth calculation is based only on the spatial offset of the intensity minima measured by two detectors. Hence, this method is independent on the contrast between defects and background and it is applicable for a wide range of optical properties and perturbations.

We have further investigated the dependence of the depth curve on the size of the inclusion. For this, we have used a background absorption coefficient (µ_a0_) of 0.05 cm^−1^, a background reduced scattering coefficient (µ_s0_’) of 5 cm^−1^, a separation between the two detector fibers (δ*D*) of 20 mm, and d_0_ = 40 mm. The inclusion was either point-like or a cube with the side of the cube ranging from 2 to 20 mm. The largest discrepancy between the *z*/*d*_0_ vs. α curves was found to yield a 2 mm difference in depth reconstruction between the point inclusion and the 20 mm inclusion. Hence, we conclude that the size of the inclusion is not expected to significantly influence the shape of the depth curve. We have also found that the curve is not sensitive to the specific values of *d*_0_ (as also reported in [22]), so that the curves of Figure 2 yield a robust tool to translate the measured offset parameter α into depth *z*.

The shallower slope of *z*/*d*_0_ versus α for mid-range values of ∼0.5 indicates a weaker sensitivity of α measurements to the depth of structures that are deeply embedded in the medium, but a more robust reconstruction of the depth. This result, which has been previously reported by using photon trajectory calculations based on diffusion theory 26,27, is complemented by the higher sensitivity of optical measurements to shallow structures. When more than one defect is present in the medium, the superposition of the contributions of the inclusions to the measured data may cause a shift of the single minima associated with each defect separately. In this case, we have found, by Monte Carlo simulations and phantom experiments, that the offset parameter α associated with two resolvable defects is the same when the two defects are individually present and when they are present at the same time in the medium. Therefore, the curve of Figure 2 that is the basis for translation of α into depth, albeit obtained for a single defect, has universal applicability to more general situations in which multiple defects are present, as long as they are resolved in the images collected by both detectors. However, two un-resolvable objects (for example two defects having the same *x* and *y* coordinates but different depths) would “obscure” each other, in at least one of the two images. In this case, the method based on the curve of Figure 2 would assign an effective depth that is in general a weighted average of the depths of the two objects, where the weights of this average depend on the location and optical contrast of the two objects.

### 2.3 Approach to Depth Discrimination

According to the basic principles described above, the key requirement for depth assessment is to measure the spatial offset parameter α associated with the locations of any detected inhomogeneity in the images collected by the detector pairs. Specifically, if we denote with **r** *_-_*_***δD***_ and **r** ***_+_***_**δ*D***_ the positions of any given object in the images collected with detectors *D_-_*_**δ*D***_ and *D_+_*_**δ*D***_, respectively, α is found by considering that α**δ** ***D*** = **r** *_-_*_**δ*D***_−**r** *_+_*_**δ*D***_. Finding the two positions **r** *_-_*_**δ*D***_ and **r** *_+_*_**δ*D***_ associated with the same object in the two images is a challenging task in the case of multiple and overlapping structures. We propose a method that builds on our breast imaging approach featuring a relatively high spatial data sampling rate (0.5 mm^−1^), further increased (to 2 mm^−1^) by data interpolation [13], and a spatial second-derivative algorithm that enhances the spatial resolution and the visualization of optical inhomogeneities [21].

The first step in our approach is to generate skeleton binary images based on second-derivative images. Using the inverse of the optical intensity data, we take the minimum of the spatial second derivatives along multiple directions (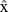, 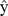, 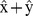, 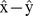). We then convert the second-derivative image into binary form by setting a value of 1 for the pixels associated with local minima of the second-derivative, and a value of 0 elsewhere. We indicate these binary images, which only retain the skeleton of geometrical shape information about the detected inhomogeneities, as *B*_-**δD**_(**x**) and *B*_+**δD**_(**x**) for the two offset paired detectors, where **x** indicates the vector for (x,y).

**Figure 3:**
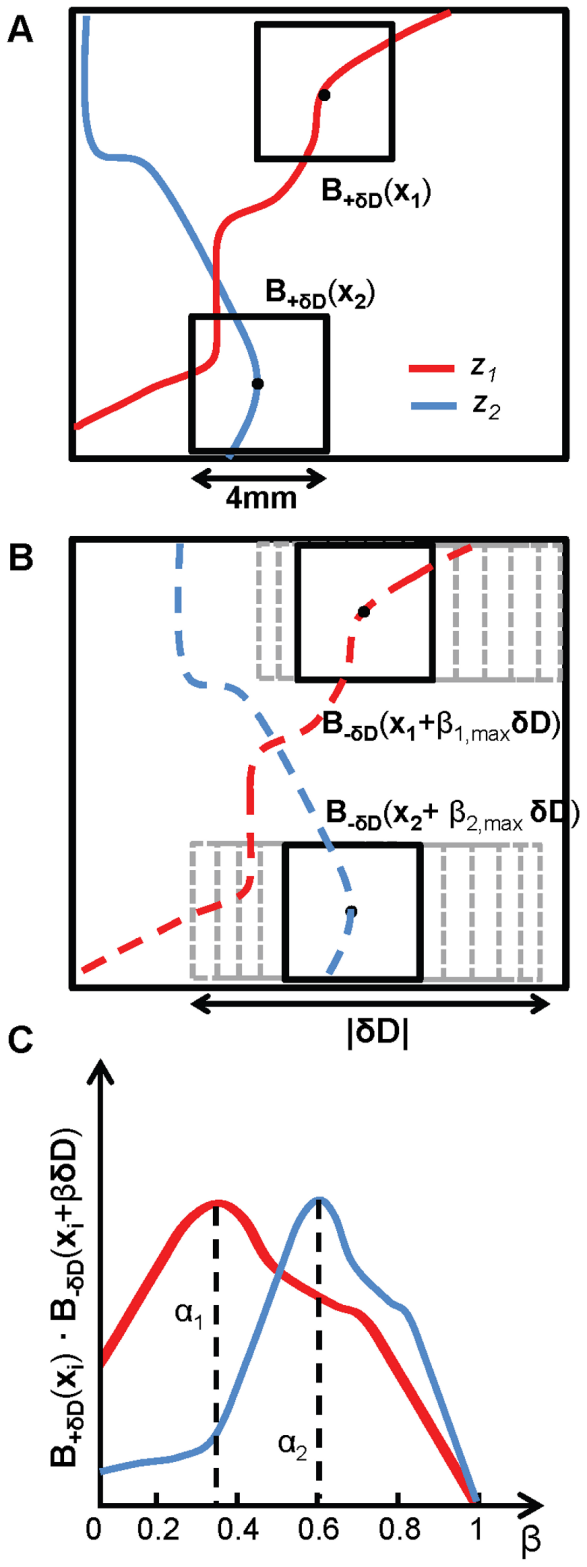
Inner product approach to depth discrimination. The approach is based on binary versions of the second-derivative images measured with the two detectors, [**B**_-δD_(**x**)] and [**B**_+δD_ (**x**)] detectors. (A) For specific inhomogeneities at **x**_1_ and **x**_2_), we consider 4 mm×4 mm windows **B**_-δD_(**x**_1_) and **B**_-δD_(**x**_2_)centered at **x**_1_ and **x**_2_, and (B) a set of 4 mm×4 mm windows shifted along **δD** by β**δD** (with 0≤ β ≤1). (C) When the inner product **B**_-δD_(**x**_i_) **B**_+δD_(**x**_i_+β_i,max_ **δD**)] shows a maximum, we set α_i_ = β_i,max_.

The second step is based on inner products of binary images *B*_-**δD**_ and *B*_+**δD**_ and a graphical representation of this second step is shown in Figure 3. For each non-zero pixel **x_i_** in the *B*_+**δD**_ binary image, we consider a square window of the *B*_+**δD**_ image centered around this pixel (**x_i_**). The size of the window must be greater than the pixel size (so that it contains at least 3×3 pixels) and smaller than δ*D* (so that multiple windows fit within the linear range 0-δ*D*). We have found that a window size of 4 mm×4 mm works well for our applications. We identify the matrix of binary data in this window centered around ***x*** **_i_** as **B**_+**δD**_(**x_i_**). Among windows of the same size in the second binary image *B*_-**δD**_ that are shifted along **δD** by no more than δD from point **x_i_**, we look for the one that is most “similar” to **B**_+**δD**_(**x_i_**). For each step, the inner product **B**_+**δD**_(**x_i_**)⋅**B**_-**δD**_(**x_i_**+β**δD**) is being calculated, where 0≤ β ≤1. We identify with β_max_ the value of β for which the inner product is maximized, and we set α = β_max_ (see Fig. 3). This inner product between two matrices of the same dimensions, sometime referred to as Frobenius inner product, is just the sum of the products of the corresponding elements of the two matrices and is an extension of the scalar product between vectors. The meaning of this maximization criterion is that it identifies the shift α**δD** for which the shape in the *x-y* plane of the inhomogeneity surrounding the point (*x*_0_,*y*_0_) is most similar in the two images collected with *D_-_*_**δ*D***_ and *D_+_*_**δ*D***_. Once the value of α that maximizes the inner product **B**_+**δD**_(**x_i_**)⋅**B**_-**δD**_(**x_i_**+α**δD**) is found, such value is associated with a depth *z* for the structure at **x_i_** (in the **B**_+**δD**_ image) by using the known relationship between *z*/*d*_0_ and α (Fig. 2).

The approach described above can be applied to multiple detector pairs with offsets along different directions **δD_i_**, therefore resulting in multiple depth measurements *z*_i_. We propose combining these multiple depth measurements by taking a weighted average where the weights are the absolute values of the spatial second derivatives of the optical intensity along direction **δD_i_**, so that depth measurements associated with detector pairs whose offset direction is perpendicular to directional structures are assigned maximum weight. For spherical objects, the weighted average reduces to a regular average. The depth measurement from *n* detector pairs is therefore given by:
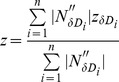
(1)where 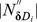 is the average, over the two-detector images, of the absolute values of the second derivative along direction **δD_i_** at the object location. For highly directional objects, such as the rods that we have used for the phantom experiments, we will show that one detector pair is sufficient for accurately determining the depth, as long as the rod is not aligned along **δD_i_**. Since the geometry of the objects imaged is typically not known, we will show that two pairs of detectors, aligned along **δD_1_** and **δD_2_**, (which are two arbitrary, not coincident directions) together with the weighted average approach described by Eq. (1), is sufficient for determining the depth of objects regardless of their orientation. For all our experiments and simulations, we have chosen **δD_i_** to be along either 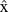 or 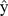.

### 2.4 Monte Carlo Simulation

**Figure 4:**
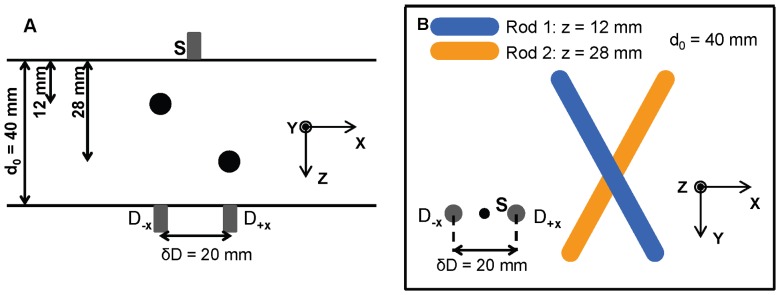
Geometrical configurations used for Monte Carlo simulations. (A) Spherical inclusions at different depths (12 and 28 mm). (B) Intersecting cylindrical inclusions located at different depths (12 and 28 mm). In both cases, the total thickness (i.e. the separation along **z** of the source S and the two detectors D_-**x**_ and D_+**x**_) is 40 mm.

A Monte Carlo method has been used for simulating different scenarios of inclusions. Details about the method have been described elsewhere [28]. In a slab with thickness *d*_0_ = 40 mm, we considered two absorbing spherical inclusions at depths *z*_1_ = 12 mm and *z*_2_ = 28 mm (Fig. 4A), or we lined two sets of ten absorbing spherical defects to simulate two intersecting rods at different depths of 12 and 28 mm (Fig. 4B). In both cases, the diameter of the inclusions was set to 6 mm. In the case of the two spherical defects, the detector scanning was linear along *x* with a scan step of 0.2 mm. In the case of the set of spheres simulating the rods, the detector scanning was planar in the *x-y* with scanning steps of 2 mm in both *x* and *y* directions. The optical properties of the background medium were µ_a0_ = 0.05 cm^−1^ and µ’_s0_ = 5 cm^−1^, while the optical inclusions behaved as highly absorbing structures, twenty times more absorbing than the background (i.e. µ_a_ = 1 cm^−1^). The contrast between the absorption of the structures and that of the background in Monte Carlo simulations was chosen to mimic the contrast between blood vessels and breast tissue.

### 2.5 Instrumentation for Experimental Tests

In order to experimentally evaluate our depth discrimination approach, phantom experiments as well as *in vivo* experiments on a female breast have been performed.

The main components of the system for phantom experiments were a near-infrared tissue oximeter (OxiplexTS, ISS Inc., Champaign, IL), a liquid phantom where the source and detector fibers of the oximeter were embedded, and a microstepper stage to control the scan of the source-detector fiber configuration. Two pairs of off axis detectors, one featuring offset along **x** and one along **y**, were used with δ*D* = 1.93 cm. Data were collected using a 690 nm laser diode light source and the step size between consecutive data points in the *x* and *y* directions was either 1 mm×1 mm or 2 mm×2 mm.

For the *in vivo* study, the system has been described in detail elsewhere [25] and shall only be summarized here. A xenon arc lamp (model no. 6258, Oriel Instrument, Stratford, CT) was used for illumination. Light transmitted through the breast thickness *d*_0_ was collected by one optical fiber bundle (5 mm in diameter) that delivered the collected light into a spectrograph (SP-150, Acton Research Corp., Acton, MA) and a charge coupled device (CCD) camera (DU420A-BR-DD, Andor Technology, South Windsor, CT). One full spectrum (wavelength range: 650–900 nm) was acquired every 2 mm along the *x* scanning coordinates. Both on-axis and off-axis scans were performed simultaneously by adding one off-axis collection optical fiber (3 mm in diameter) which delivered light to a photomultiplier tube detector (R928, Hamamatsu Photonics, Hamamatsu City, Japan). The off-axis collection optical fiber was offset along -**x** with δ*D* = 1.3 cm. Successive scanning lines along the *y* coordinate were separated by 2 mm, resulting in a square pixel size of 2 mm×2 mm for the collected images.

### 2.6 Tissue-like Liquid Phantom

**Figure 5:**
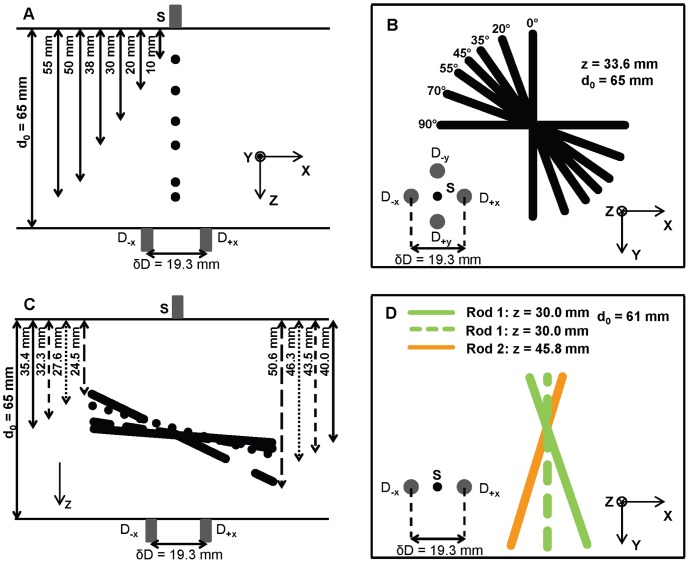
Setup for the liquid phantom experiments. The inclusion used was a black rod of 3.5 mm diameter. The rod was varied in depth (A), rotated in the x-y plane (B), tilted in x-y and z direction (C). A second rod was also used, at a different depth than the first rod, and intersecting the other one on a projection on the x-y plane (D).

The liquid phantom consisted of 2% fat milk diluted in water (volume ratio: 3∶5) and contained in a tank (size: 40 cm×20 cm×25 cm), to mimic an infinite medium geometry with the source and detection fibers deeply immersed inside the liquid phantom. The optical properties of this medium were described by an absorption coefficient µ_a0_ ∼ 0.005 cm^−1^ and a reduced scattering coefficient µ’_s0_ ∼ 7 cm^−1^ at a wavelength of 690 nm. For inclusions, black plastic cylinders with a diameter of 3.5 mm were used. The orientation, size, as well as the depth of the rods can be seen in Figure 5. Single rods were used for evaluating the accuracy of depth calculation at different depths (Fig. 5A). In order to evaluate whether there is a dependence on the angular orientation of the rod, the rod has been rotated in the *x*-*y* plane within the same depth and scans have been performed with two off-axis detectors in the **x** as well as **y** directions (Fig. 5B). In addition to tilting the rod in the *x*-*y* plane, we also tilted it in z as shown in Figure 5C. Lastly, two rods at different depths (30 mm and 45.8 mm) with intersecting projections on the *x*-*y* plane have been used (Fig. 5D) with rod 1 making an angle of either 0 or 30 degrees with respect to the positive *y* axis, and rod 2 making an angle of –20 degrees with respect to the positive *y* axis. Since the absorption coefficient of the phantom used was that of water, the specific depth curve for µ_a0_ = 0.005 cm^−1^ and µ’_s0_ = 5 cm^−1^ in Figure 2 was used.

### 2.7 Vessel-like Structures

**Figure 6:**
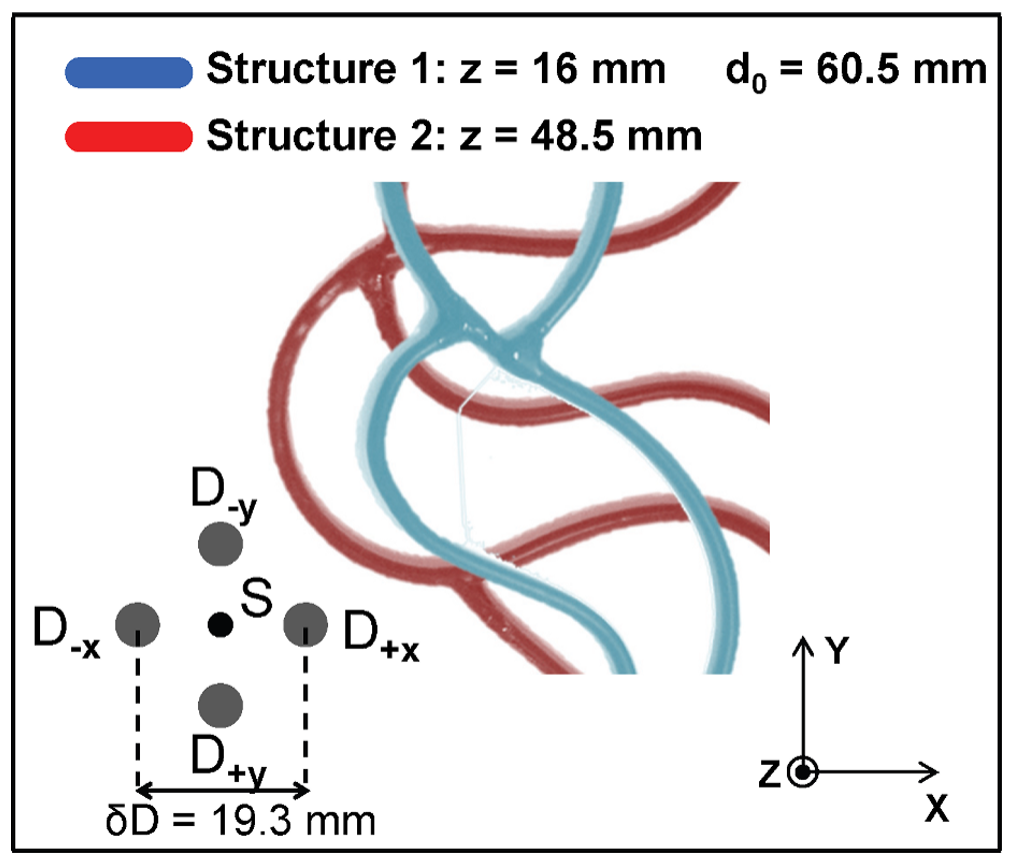
Vessel like structures. For mimicking more complex structures, black rods, shaped in a more vessel like fashion, have been used. Experiments were run with the deeper structure alone as well as both together.

Because we are specifically targeting applications to optical mammography, and since we have shown previously that inclusions such as blood vessels can be visualized [21], [25], more realistic vessel structures have been used as inclusions as well, which were made out of flexible metal wires covered with black tape. The two structures can be seen in Figure 6 (false color), each at a different depth. Again, scanning was performed with two pairs of off axis detectors featuring offsets along **x** and **y**, respectively. Images were taken with an individual structure at a depth *z* = 48.5 mm, and with two structures at depths *z*_1_ = 16 mm and *z*_2_ = 48.5 mm.

### 2.8 Human Subject

The human subject was a 20-year-old healthy Caucasian female. The subject’s right breast was placed between two glass plates, which made contact with the breast by applying a minimal compression to guarantee a stable breast position without causing discomfort to the subject. The scan of the entire breast, with simultaneous collection of one on-axis and one off-axis detector aligned with the *x* direction, took approximately 3.5 min. The data were analyzed using the depth curve obtained with background optical properties of µ_a0_ = 0.05 cm^−1^ and µ’_s0_ = 10 cm^−1^ (Fig. 2).

## Results

### 3.1 Monte Carlo Simulation Results

**Figure 7:**
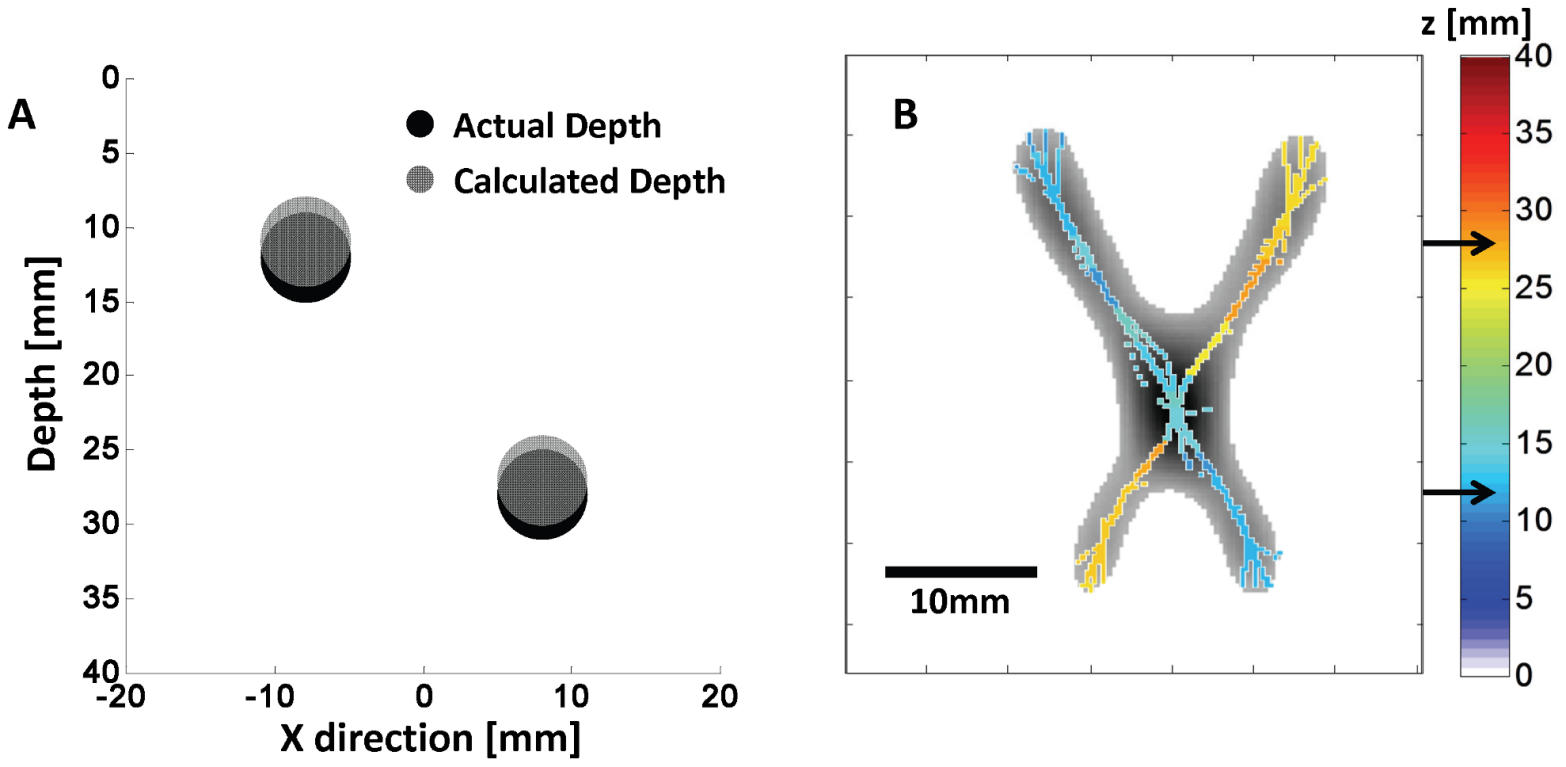
Results from Monte Carlo simulation. (A) The actual depth (black spheres at depths of 12 and 28 mm) of the spherical inclusions can be accurately determined (grey spheres at depths of 10.9±0.6 mm 27.1±0.3 mm). (B) The depth of cylindrical inclusions can also be accurately recovered for every point in the x-y plane where they do not overlap, whereas in the overlap region the cylinder closer to the source dominates the depth assessment. The black arrow in the depth color bar indicate the actual depths of the two spheres (panel (A)) and two cylinders (panel (B)).

The Monte Carlo simulation results are reported in Figure 7. Figure 7A shows that the depths of the spherical inclusions were recovered with good accuracy, with *z*_1_ = 10.9±0.6 mm (actual value: 12 mm) and *z*_2_ = 27.1±0.3 mm (actual value: 28 mm). The error was defined by assuming an inaccuracy of one pixel (0.2 mm) in determining the offset parameter α. For the scanning step of 0.2 mm used in the case of Figure 7A, the error was found to be ten times smaller than the inclusion diameter. Figure 7B shows the second derivative image obtained with D_-**δD**_ (grey scale) in the case of the two intersecting rods. Overlaid on the second derivative image is the pixel wise reconstructed depth of the rods (color scale). The average depths of the rods are *z*_1_ = 13.3±1.5 mm and *z*_2_ = 26.6±0.9 mm, respectively. At the intersection of the rods, the depth measurement yields the depth of the upper rod (which is closer to the source), indicating that the upper rod dominates the depth assessment because of the greater intensity contrast associated with it. Black arrows in the color bar indicate the actual depths.

### 3.2 Experimental Test on the Tissue-like Phantom

**Figure 8:**
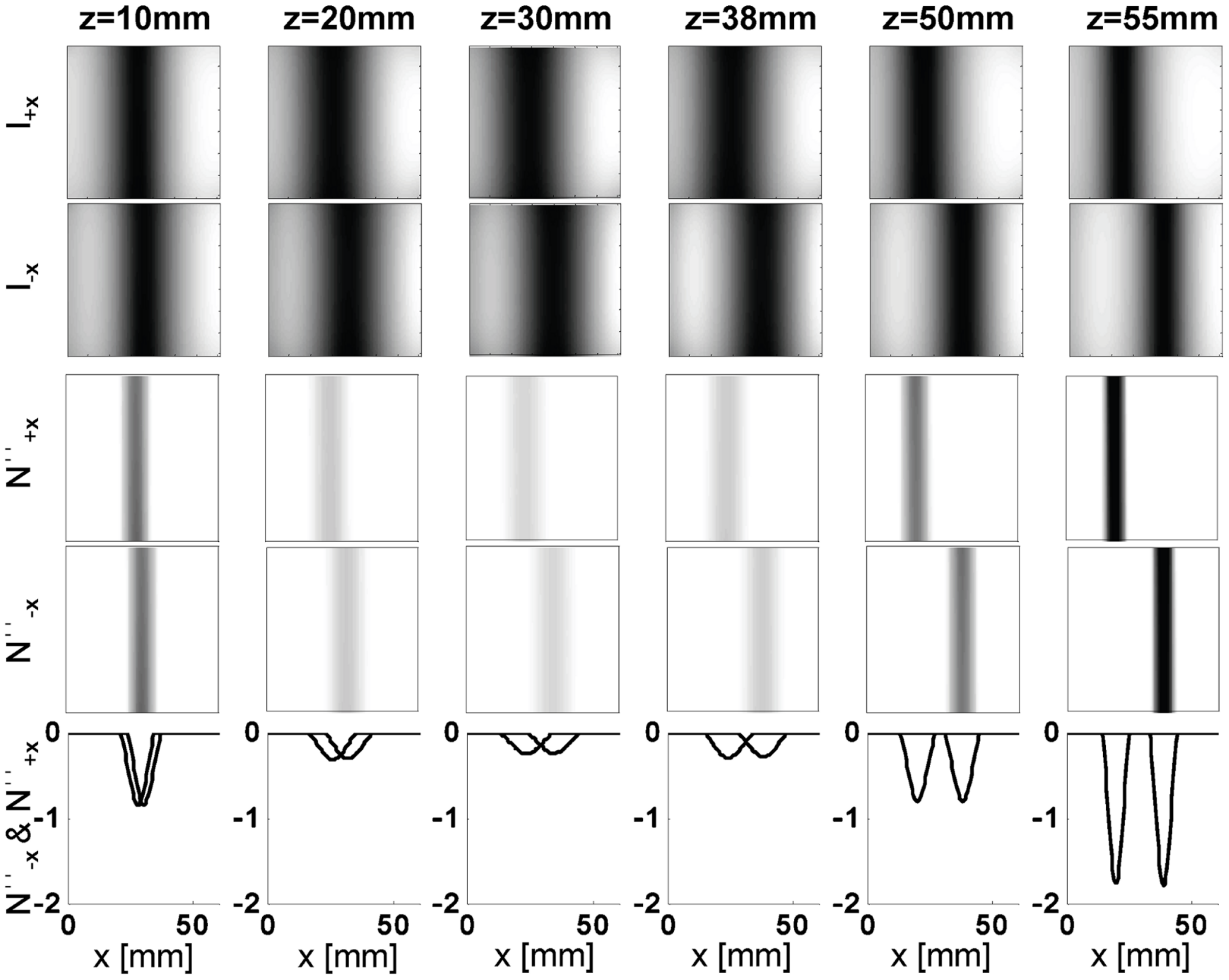
Experimental results from a rod inclusion at different depths. The figure illustrates clearly the offset between the location of the detected rod in the images collected with the two detectors, and how it increases with the depth of the inclusion.

The images in the first and second rows of Figure 8 are the raw intensity optical images (I_+**δD**_ and I_-**δD**_) for off-axis detectors D**_+x_** and D**_-x_**, respectively, associated with the rod depths indicated on the top of the images according to the geometry of Figure 5A. These images show that for rods that are closer to the detector (greater *z* values), the rod locations in the two image are shifted by a greater amount along *x* (greater α) in comparison to rods that are closer to the source (low *z* values). Taking the spatial second derivative of the raw intensity images yields the *N*′′ images shown in rows three and four. A cross section through the second derivative images for both detectors is seen in row five. In this ideal case, the offset parameter α can be easily measured from the shift along *x* between the valleys measured with the two off-axis detectors.

**Figure 9:**
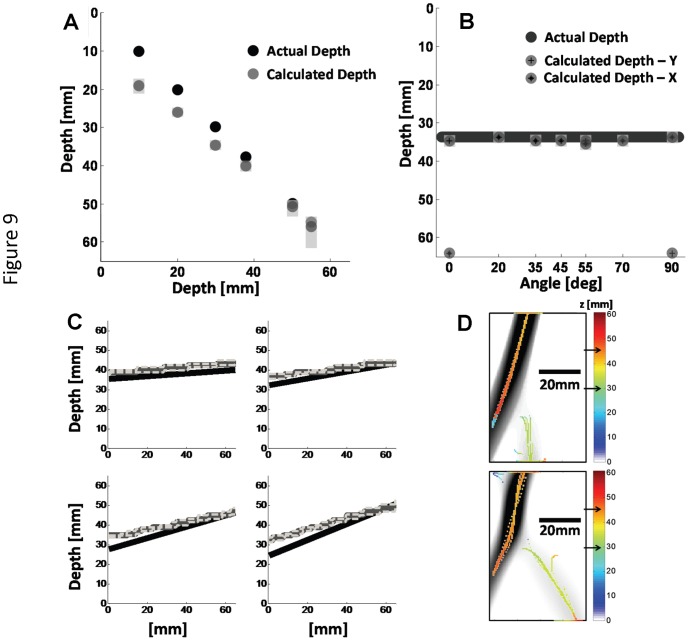
Experimental results from the rod inclusions seen in **Figure 5**. In 9A and 9B, the actual depth is shown in black and the measured depths are shown in dark grey with error bars in light grey. (C) shows the tilted rods, where the actual rod is shown in black, the measured one is displayed with the thick grey line and the depth dependent error by the thin grey lines. (D) shows the second derivative image of D_-**x**_, overlaid with the pixelwise calculated depth.

The conversion of α into the actual depth can be seen in Figure 9A for each depth location of the rod. These calculated depths (grey circles) were found to be within 4 mm of the actual depth for a rod in the center of the phantom and within 7 mm for a rod close to the surface in comparison to the actual depths (black circles). The error bars were calculated by assuming a 1 pixel (0.5 mm) uncertainty in measuring the offset between the rod locations measured with the two detectors. Since the depth curve shown in Figure 2 is steeper closer to the source and the detector, the calculated error is larger in those regions in comparison to the center of the phantom.

Figure 9B shows the results for the angled rods, which demonstrate that the orientation of the rod relative to the **δD** direction (which is along either *x* or *y* in this experiment) does not influence the measured depth, as long as the rod is not aligned with **δD** (i.e., rod axis at 0 degrees for **δD** along *x*, and 90 degrees for **δD** along *y*). In Figure 9B, the two sets of dots representing the depth measurements based on the detector pairs offset along *x* and *y* are coincident for all rod directions, except for the cases in which the rod is oriented along *x* or *y*. Results for tilting the rod in both the *x*-*y* plane and in depth can be seen in Figure 9C, with results plotted against the **x** direction. The four plots refer to the four cases illustrated in Figure 5C. Again, we found that the rod direction in the *x*-*y* plane does not affect the results. For the cases when there are two structures with intersecting projections on the *x-y* plane, the depth of each rod can be measured accurately, except for the location of the intersection. For the location of the intersection, only one depth is recovered, and in Figure 9D such depth is *z* = 45.8mm, which corresponds to the rod that is farther from the mid-depth plane and is thus associated with the greater optical contrast. The two images in Figure 9D refer to the two cases illustrated in Figure 5D. The arrows in the colorbar indicate the actual depth.

### 3.3 Vessel-like Structures

**Figure 10:**
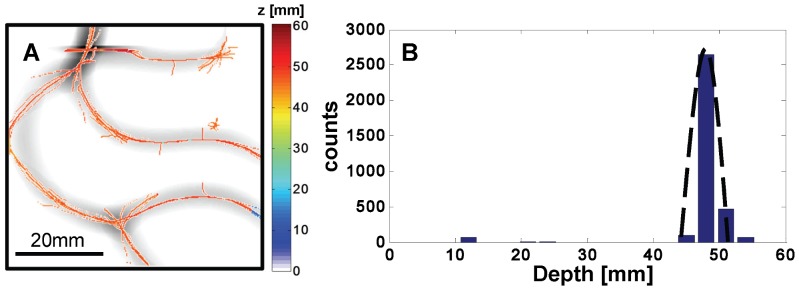
Experimental result of the vessel-structured rods located at depth z = 48.5 mm. (A) shows the averaged second derivative image overlaid with the recovered depth values. The histogram of the calculated depths for all pixels of the structure can is shown in (B). The dashed line is a Gaussian centered at the actual location of the rod.

**Figure 11:**
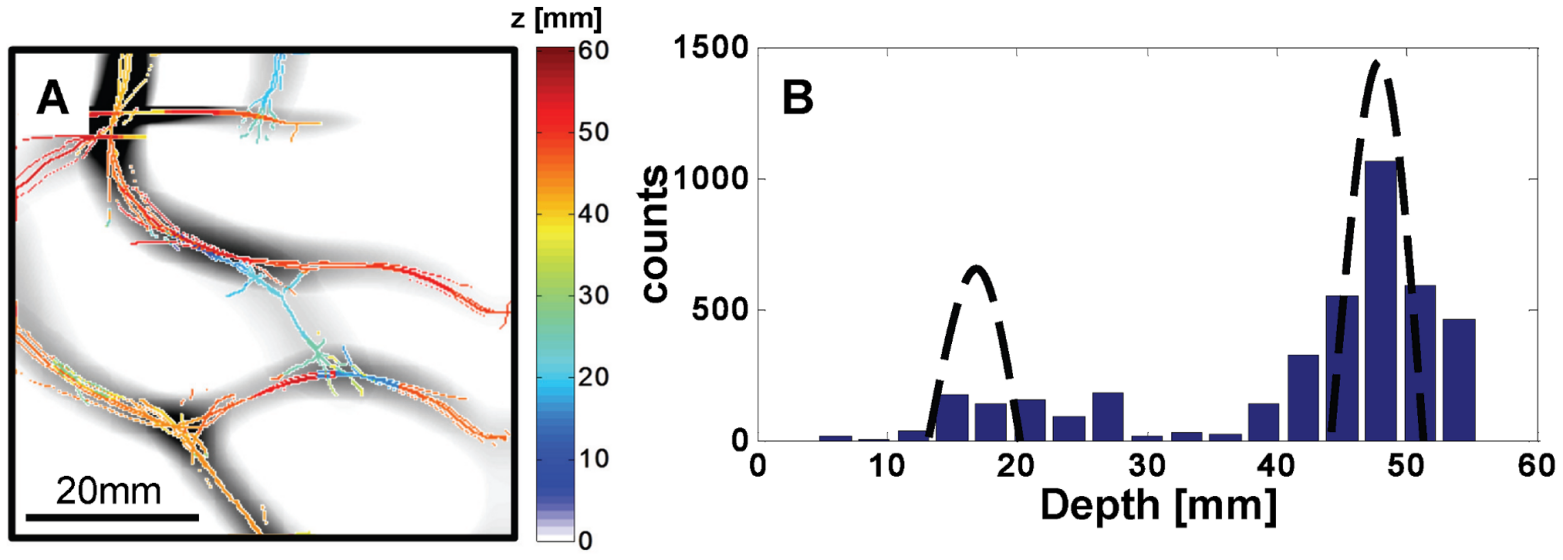
Experimental result of the vessel-structured rods located at depths z_1_ = 16 mm and z_2_ = 48.5 mm. (A) shows the averaged second derivative image overlaid with the recovered depth values. The histogram of the calculated depths for all pixels of the structure is shown in (B). The dotted bell curve corresponds to the actual depth location and the width to the error in depth based on 1 pixel uncertainty in finding the value of α**δD**.

In the case of more complicated, vessel like structures, images have been taken with the deeper structure alone (*z* = 48 mm) (Fig. 10) as well as with both structures, each at a different depth (z_1_ = 16 mm and z_2_ = 48.5 mm) (Fig. 11). Figure 10A shows the average second derivative image of the two off axis detectors D_-**x**_ and D_-**y**_ (detector pairs being aligned along x and y, respectively). Superimposed on the second derivative image are the color-coded depth values calculated according to Eq. (1). Multiple structures are present in the *x-y* projection images and the determination of the offset parameter α is not easily done because the identification of corresponding structures in the images collected with the offset detectors is not straightforward as in the case of individual inhomogeneities. In order to assign the value α to each pixel associated with an optical inhomogeneity, we have used the procedure of maximizing the inner product **B**_+**δD**_(**x_i_**)⋅**B**_-**δD**_(**x_i_**+β**δD**) to find α as described in section 2.3. The histogram of all calculated depths can be seen in Figure 10B. The dashed line is a Gaussian centered at the actual location of the rod with a standard deviation given by assuming one pixel (0.5 mm) error in finding the value of α.

Figure 11 reports the case in which there are two structures located at two different depths. In Figure 11A, the average second derivative image is overlapped with the color-coded depth measurements. For comparison with the known depths, the histogram in Figure 11B shows two peaks that are centered around the actual depths *z*_1_ = 16 mm and *z*_2_ = 48.5 mm. The center of the dotted bell curve corresponds to the actual location of the rod and the width to the error in depth based on one pixel uncertainty in finding the value of α. The peaks in the histogram for the shallower structure (blue in Fig. 11A) are smaller compared to the deeper structure (red in Fig. 11A) because the shallower structure was smaller in terms of surface area and is therefore associated with a smaller number of pixels in the *x-y* images.

### 3.4 Measurements on the Human Breast

**Figure 12:**
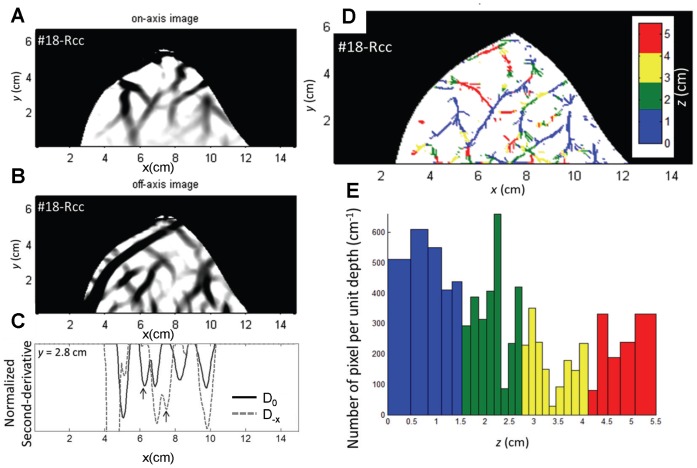
Depth discrimination results of the human study [subject #18, right breast measured in cranio-caudal projection (Rcc)]. The starting point consists of the second-derivative, 2D projection images obtained with the on-axis (panel (A)) and off-axis (panel (B)) detectors. (C) Second-derivative lines at y = 2.8 cm for on-axis (continuous line) and off-axis (dashed line) acquisition, with two paired peaks indicated by the vertical arrows. (D) Depth-resolved image of the examined female breast, with color-coded depth representation for all detected inhomogeneities. (E) Histogram built on the 27 depth layers resolved over the 5.5 cm breast thickness. The y axis represents the pixel density, i.e. the number of pixel corresponding to a given depth bin, per unit depth.

In the case of the human breast measurements, one detector fiber was collinear with the illumination fiber and was therefore indicated with D_0_ according to our notation convention. Figure 12A and 12B show the second derivative images associated with D_0_ and D_-**x**_, respectively, measured on the female breast. A number of structures, which for the most part we assign to blood vessels, are visible in both images. Representative second-derivative lines as a function of *x*, at a fixed *y* coordinate of 2.8 cm, are shown in Figure 12C and illustrate the complex task of pairing on-axis peaks (continuous line) with the corresponding off-axis peaks (dashed line). Our method based on the inner product of skeleton images assigns the first of the two on-axis peaks at 6 cm<*x* <7 cm to the second of the two off-axis peaks at 7 cm<*x* <8 cm, as indicated by the arrows in Figure 12C. By applying the inner product approach to all second-derivative minima, we obtain an image of the depth of all detected inhomogeneities in the breast, as shown in Figure 12D. The corresponding histogram of the depth distribution is shown in Figure 12E, where the variable bin size reflects the fact that the sensitivity of α on *z* is different at different depths (see Fig. 2). To take this into consideration, we plot the number of pixels per unit depth on the ordinate of the histogram, so that the area of each bar represents the number of pixels associated with depths within the corresponding *z* bin. In Figures 12D and 12E, we color-code the depth values to highlight superficial structures (blue: 0 cm<*z* <1.5 cm; red: 4.1 cm<*z* <5.5 cm), and deeper structures (green: 1.5 cm<*z* <2.8 cm; yellow: 2.8 cm<*z* <4.1 cm). According to this classification, about 63% of the pixels are associated with superficial structures, while the remaining 37% are associated with deeper structures.

## Discussion

In this work, we have reported a proof of principle demonstration for a novel approach to depth discrimination in planar diffuse optical imaging. For such demonstration, we have used a pair of optical detectors that are offset by δ*D* along direction **δD** in the scanning plane *x-y*. As a result, detected inhomogeneities appear shifted (by an amount within the range 0-δ*D*) along direction **δD** in the images acquired with the two optical detectors. By applying this approach using detector pairs shifted along multiple directions **δD_i_**, one can enhance the depth assessment of directional structures by taking a proper weighted average of the depths recorded by the multiple detector pairs (see Eq. (1)). In comparison to 3D tomography where depth discrimination is possible, even potentially with the same high spatial sampling rate, the presented approach is robust in the sense that depth information is based on a look up table (depth curves in Figure 2) and no assumptions are necessary. The disadvantage in comparison to 3D tomography is found when structures are overlapping or intersecting. In this case, the method will only assign one depth.

The depth resolution is mostly affected by the offset δ*D* between the two detectors. The greater δ*D* the better the depth resolution. However, a large offset δ*D* may introduce artifacts close to the sample edge as one of the optical fibers may get beyond the sample edge, and can increase the number of inhomogeneities that do not appear in both images collected with D_-**δD**_ and D_+**δD**_. Furthermore, a large δ*D* may also result in different shapes of a given inhomogeneity, as projected on the *x-y* plane, in the images collected with the two paired detectors. This can be problematic, because the similarity of the *x-y* projections of detected structures in the two detector images is the basis for pairing them up according to our inner-product method. Furthermore, a large δ*D* would also increase the source-detector distance, thus reducing the signal-to-noise ratio for that measurement. We have found that the values of δ*D* used in the phantom experiment (1.93 cm) and in the human experiment (1.3 cm) achieve a good compromise between yielding a good depth resolution and allowing for robust pairing of corresponding structures in the two detector images. We point out again that the depth resolution is not uniform along the axial coordinate *z* as a result of the non-linear relationship between the offset parameter α and the object depth *z* (see Fig. 2).

Our approach for pairing corresponding structures in the two detector images is based on the inner product of data windows from the skeleton binary images, the first one from the D_+**δD**_ image centered around the pixel of interest **x_1_**, and the second one from the D_-**δD**_ image centered around a pixel shifted from **x_1_** by 0-δ*D* along direction **δD** [**B**_+**δD**_(***x*** **_i_**)⋅**B**_-**δD**_(***x*** **_i_**+β**δD**), 0≤ β ≤1]. Where the inner product is maximized, α = β_max_. The size of such data windows should not be too large so not to include extraneous additional structures, and not too small to include a good portion of the structure surrounding the pixel of interest **x_1_**. We found that the size 4 mm×4 mm achieves a good compromise between these requirements for the phantom and human studies reported here, but a different size may be appropriate for different samples, different sample thicknesses *d*_0_, or a different detector offset δ*D*.

We conclude by noting that, in the human study, we have not been able to assign a value of α to about 9% of the pixels associated with optical inhomogeneities in the on-axis image of Figure 12A. The reason is that, for these pixels, the inner product **B**_+**δD**_(***x*** **_i_**)⋅**B**_-**δD**_(***x*** **_i_**+β**δD**) was 0 for every value of β. This problem, even though it only affected 9% of the pixels in our case, can be at least partially solved by using additional off-axis detector optical fibers along different directions, as shown in phantom experiments (Figs. 10 and 11).

### Conclusions

We have reported a novel approach to depth discrimination for diffuse optical imaging in a parallel plate configuration. At the heart of the approach, there is (1) data collection with two detection fibers shifted by δ*D* in the direction **δD**, (2) an inner-product based method for pairing detected inhomogeneities in the two detector images, and (3) a weighted average of the depth measurements with detector pairs along multiple directions **δD_i_**. This approach offers the advantage of being robust even in the presence of multiple detected inhomogeneities as one would expect to be the case when imaging biological tissues. In particular, we have demonstrated the applicability of our proposed approach to optical mammography. In conjunction with previously demonstrated oximetry capabilities [13], [25], this depth discrimination approach has the potential to achieve an oximetric 3D rendering of optical inhomogeneities in the female breast, which can translate into more effective detection of breast cancer and/or monitoring of response to neoadjuvant chemotherapy.
